# Antitumor Peptides from Marine Organisms

**DOI:** 10.3390/md9101840

**Published:** 2011-10-10

**Authors:** Lan-Hong Zheng, Yue-Jun Wang, Jun Sheng, Fang Wang, Yuan Zheng, Xiu-Kun Lin, Mi Sun

**Affiliations:** 1 Yellow Sea Fisheries Research Institute, Chinese Academy of Fishery Sciences, Qingdao 266071, China; E-Mails: zhenglanhong@126.com (L.-H.Z.); wangyj@ysfri.ac.cn (Y.-J.W.); shengjun01@hotmail.com (J.S.); wendywf2002@yahoo.com.cn (F.W.); zhengyuan@ysfri.ac.cn (Y.Z.); 2 Department of Pharmacology, Capital Medical University, Beijing 100069, China; 3 Institute of Oceanology, Chinese Academy of Science, Qingdao 266071, China

**Keywords:** antitumor, peptides, marine organisms

## Abstract

The biodiversity of the marine environment and the associated chemical diversity constitute a practically unlimited resource of new antitumor agents in the field of the development of marine bioactive substances. In this review, the progress on studies of antitumor peptides from marine sources is provided. The biological properties and mechanisms of action of different marine peptides are described; information about their molecular diversity is also presented. Novel peptides that induce apoptosis signal pathway, affect the tubulin-microtubule equilibrium and inhibit angiogenesis are presented in association with their pharmacological properties. It is intended to provide useful information for further research in the fields of marine antitumor peptides.

## 1. Introduction

The sea, covering 70% of the Earth’s surface, offers a considerably broader spectrum of biological diversity than *terra firma*. Containing approximately 75% of all living organisms, the marine environment offers a rich source of natural products with potential therapeutic application. The discovery of the bio-regulatory role of different endogenous peptides in the organism as well as the understanding of the molecular mechanisms of action about some new bioactive peptides obtained from natural sources on specific cellular targets, contributed to developing peptides as promising lead drug candidates. Recently, marine peptides have opened a new perspective for pharmaceutical developments [1].

**Figure 1:**
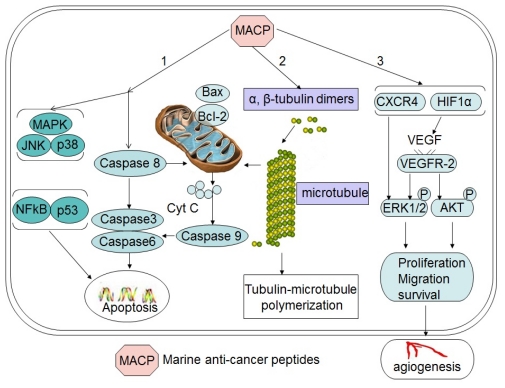
Schematic depiction of major mechanisms for major antitumor peptides. Marine peptides induce cell death via the following pathways, apoptosis (1), affecting the tubulin-microtubule equilibrium (2) and angiogenesis pathway (3).

Peptides discovered from marine organism induce cell death with different mechanisms, including apoptosis, affecting the tubulin-microtubule equilibrium, or inhibiting angiogenesis (Figure 1). This finding has increased our knowledge about new potent cytotoxic, and many other properties with novel chemical structures associated to original mechanisms of pharmacological activity. These facts introduce marine peptides as a new choice for obtaining lead compounds on biomedical research. This review will account for the progress of the recent years and provide novel information in this field.

## 2. Peptides that Induce Apoptosis

Apoptosis as a form of programmed cell death is one of the major mechanisms of cell death in response to cancer therapies [2]. Also, apoptosis is a naturally occurring and evolutionarily conserved process by which cells that are no longer useful are directed to their deaths [3]. Apoptosis plays an indispensable role and is a fundamental process in development, physiology and homeostasis [4–7]. Its deregulation, *i.e.*, either loss of pro-apoptotic signals or gain of anti-apoptotic signals, can lead to a variety of pathological conditions such as cancer initiation, promotion and progression or result in treatment failures [8,9]. As apoptosis does not usually trigger inflammatory or immune response, it becomes a preferable way of cancer cell death during cancer treatments. As such, modulation of apoptotic pathways and selective induction of apoptosis by chemical agents are likely to be a promising approach for cancer therapy [6,10–15]. In mammals, there are two major signaling systems that result in the activation of caspases, the extrinsic death receptor pathway [16–19] and the intrinsic mitochondrial pathway [20,21]. These two pathways include many cross talks between them. There is a long list of pro- or anti-apoptotic molecules that can trigger or regulate apoptosis. Therefore, developing anticancer peptides that target these molecules has become an important strategy for anticancer therapies.

Growing evidence shows that most marine anticancer peptides with cytotoxicity may trigger apoptosis by targeting many cellular proteins, and the induced apoptotic process involves both intracellular and extracellular pathways [14]. The balance between the pro-survival gene Bcl-2 and the pro-apoptotic gene Bax plays a key role in maintaining cell viability. Therefore, inhibition of Bcl-2 or induction of Bax becomes a good strategy for triggering an apoptotic process [22]. Identification of caspases activators becomes another approach for the discovery of novel anticancer agents because caspases are involved in intrinsic and extrinsic apoptosis pathways [23,24]. Some marine anticancer peptides can activate the Jun *N*-terminal kinase (JNK) and p38 mitogen-activated protein kinases (MAPK) pathways that lead to the release of cytochrome c (Cyt C) from mitochondria [25]. In brief, apoptosis is a complicated process and involves a huge number of signaling molecules, and failure of apoptosis activation is one of the major impediments to the treatment of cancer. Therefore, a good strategy for the development of new anticancer agents is to identify or develop such agents that can target multiple apoptosis regulating genes.

### 2.1. Peptides that Activate the Intrinsic Mitochondrial Pathway

The mitochondrial cell death pathway commences when apoptogenic molecules presented between the outer and inner mitochondrial membranes are released into the cytosol by mitochondrial outer membrane permeabilization. The release of pro-apoptotic factors such as cytochrome c from mitochondria leads to formation of a multimeric complex known as the apoptosome and initiates caspases activation cascades. These pathways are important for normal cellular homeostasis and play key roles in the pathogenesis of many diseases [21,26]. In the intrinsic mitochondrial pathway, at least 18 pro- and anti-apoptotic proteins of the Bcl-2 family are pivotal regulators of apoptosis, Bax as a proapoptotic member of the Bcl-2 family is very influential in the pro- and anti-apoptotic balance by regulating mitochondrial functions [22,27].

**Figure 2:**
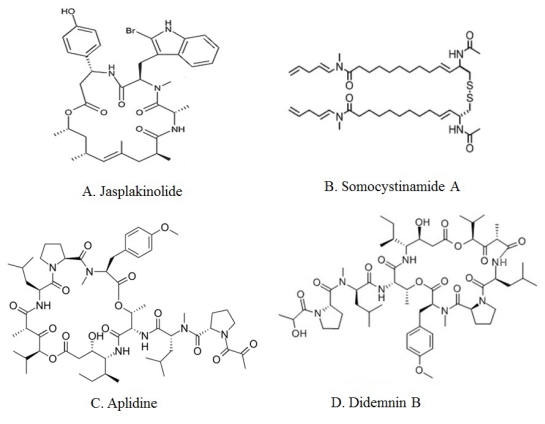
Chemical structures of major marine peptides with apoptotic activity: Jasplakinolide (**A**), Somocystinamide A (**B**), Aplidine (**C**) and Didemnin B (**D**).

Jaspamide (Jasplakinolide), isolated from marine sponge *Jaspis johnstoni*, is a cyclic depsipeptide with a 15-carbon macrocyclic ring containing three amino acid residues (Figure 2A) [28]. Jasplakinolide induces apoptosis in Jurkat T cells as demonstrated by nucleosomal DNA ladder formation. Enhanced caspase-3 activity is also observed in Jaspamide-treated Jurkat T cells by using the fluorescent substrate DEVD-MAC. Transformed cell lines were more susceptible to Jasplakinolide-induced apoptosis than normal nontransformed cells [29]. Jaspamide-induced apoptosis is associated with caspase-3 activation and a decrease in Bcl-2 protein expression, but also with increased Bax levels. It seems that jaspamide induces a caspases independent pathway of cell death, which is responsible for the observed cytoplasmic and membrane changes in apoptosing cells, and also a caspases-dependent cell death, which is responsible for PARP proteolysis [30].

Some other peptides from marine sources, such as Somocystinamide A [31,32] and *C*-phycocyanin (*C*-PC) [33,34] have been observed to display potent caspases-dependent anti-apoptotic activity in different cancer cells. Somocystinamide A (ScA), a lipopeptide, was isolated from *Lyngbya majuscula*/*Schizothrix* sp. assemblage of marine cyanobacteria (Figure 2B) [31]. ScA stimulates apoptosis in a number of tumor cell lines and in angiogenic endothelial cells via both the intrinsic and extrinsic pathways, but the more effective mechanism is the activation of caspase-8 and its downstream pathways [32]. *C*-phycocyanin, a tetrapyrrole-protein complex isolated from the cyanobacteria *Agmenellum quadruplicatum*, *Mastigocladus laminosus* [33] and *Spirulina platensis*, could induce the activation of pro-apoptotic gene and down-regulation of anti-apoptotic gene expression, then facilitate the transduction of apoptosis signals that result in the apoptosis of HeLa cells *in vitro*. Caspases 2, 3, 4, 6, 8, 9, and 10 were activated in *C*-PC-treated HeLa cells, suggesting that *C*-PC-induced apoptosis was caspases-dependent. *C*-PC treatment of HeLa cells also results in release of cytochrome c from the mitochondria into the cytosol that was related to apoptosis of *C*-PC-treated HeLa cells [34].

### 2.2. Peptides that Target the JNK or p38 MAPK Pathway

Jun *N*-terminal kinases (JNKs) and p38 mitogen-activated protein kinases (MAPKs) play critical roles in the signaling mechanisms that orchestrate cellular responses to various types of cellular stress [35,36]. Unscheduled proliferation is a hallmark of cancer, and the JNK and p38 MAPK pathways regulate cell cycle progression at different points by both transcription-dependent and transcription-independent mechanisms, with profound effects on the development of various cancers. The pro- and anti-apoptotic effects of JNKs seem to be dependent not only on the stimuli, but also on the strength of the signals. Activation of the JNK and p38 MAPK pathways can trigger cytochrome c release and subsequently activate caspases cascades [35].

Aplidine (dehydrodidemnin B, DDB, Aplidin), a cyclic depsipeptide (Figure 2C), was isolated from the Mediterranean tunicate *Aplidium albicans*. Breast, melanoma and non-small-cell lung cancer appear to be sensitive to low concentrations of Aplidine [37,38]. Aplidine’s mechanism of action involves several pathways, including cell cycle arrest, inhibition of protein synthesis. Aplidine induces early oxidative stress and results in a rapid and persistent activation of JNK and p38 MAPK phosphorylation with activation of both kinases occurring very rapid, long before the execution of apoptosis, and full activation within 5–10 min of drug treatment in human HeLa tumor cells. JNK and p38 MAPK activation results in downstream cytochrome c release and activation of caspases-9 and -3 and PARP cleavage, demonstrating the mediation of the mitochondrial apoptotic pathway in this process. Protein kinase C delta (PKC-d) mediates the cytotoxic effect of Aplidin and that it is concomitantly processed and activated late in the apoptotic process by a caspases mediated mechanism [39]. Aplidin induces apoptosis in MDA-MB-231 breast cancer cells, resulting in sustained activation of the epidermal growth factor receptor (EGFR), the non-receptor protein-tyrosine kinase Src, and the serine/threonine kinases JNK and p38 MAPK. Two mechanisms by which Aplidin activates JNK: rapid activation of Rac1 small GTPase and down regulation of MKP-1 phosphatase. Aplidine, also called plitidepsin in clinical trials, is well-tolerated with minor toxicity in finished Phase I clinical trials [40–42]. Phase II studies are underway [43].

### 2.3. Peptides with an Unknown Mechanism of Apoptosis-Inducing Activity

Some marine peptides are known to induce cell death with apoptotic characteristics, including DNA fragmentation, nucleic shrinking and cell membrane swelling. However, the exact mode of action of cytotoxicity is unclear. Didemnins, originally reported in 1981 [44,45], are a family of depsipeptides with antitumor, antiviral and immunosuppressive activities primarily isolated from the Caribbean tunicate *Trididemnum solidum*, but later obtained from other species of the same genus [46,47]. Didemnin B, a branched *N*-methylated cyclic peptolide, originally was isolated from the *Trididemnum* genus of marine tunicates (Figure 2D). Didemnin B induces death of a variety of transformed cells with apoptotic morphology and DNA fragmentation within the cytosol and the generation of DNA ladders [48], but the exact mechanism for these effects is still obscure [49]. Until today, a lot of Didemnin analogues have been prepared semisynthetically and their biological activities evaluated, including cytotoxicity and antiviral and immunosuppressive properties [46,50]. However, Didemnin B continues to be a main focus in clinical investigation, being the first marine natural product currently in clinical trials as an anti-cancer agent [51,52].

Some other peptides from marine sources, such as Sansalvamide A [53,54], Cycloxazoline [55,56] and virenamides A–C [57] have been observed to display potent anti-apoptotic activity in different cancer cells but the exact targets by these chemicals have not yet been identified. Sansalvamide A, a cyclic depsipeptide produced by a marine fungus, has demonstrated significant anticancer activity [54]. One of Sansalvamide A analogs caused G (1) phase cell cycle arrest in two human pancreatic cancer cell lines (AsPC-1 and CD18); Sansalvamide A, an inhibitor of topoisomerase I, induces cell death with only some apoptotic characteristics in some cancer cells [53]. Cycloxazoline, a new cyclic hexapeptide is reported from a marine ascidian *Lissoclinum bistratum* [55]. Accumulation of HL-60 leukemia cells in G2/M and inhibition of cytokinesis was caused by cycloxazoline [56]. Three new linear cytotoxic tripeptides, virenamides A–C have been isolated from didemnid ascidian *Diplosoma virens* [57]. The virenamides showed modest cytotoxicity towards a panel of cultured cells: Virenamide A gave IC_50_ of 2.5 μg/mL against P388, and 10 μg/mL against A549, HT29 and CV1 cells, and exhibited topoisomerase II inhibitory activity. Virenamides B and C both gave IC_50_ of 2.5 μg/mL against P388, A549, HT29, and CV1 cells [57].

## 3. Peptides that Affect the Tubulin-Microtubule Equilibrium

Microtubules are intracellular organelles formed from the protein tubulin. These organelles have a number of essential cellular functions including chromosome segregation, the maintenance of cell shape, transport, motility, and organelle distribution. Drugs that affect the tubulin-microtubule equilibrium are effective anticancer drugs [58]. Tubulin binding molecules have generated considerable interest after the successful introduction of the taxanes into clinical oncology and the widespread use of the *Vinca* alkaloids Vincristine and Vinblastine. These compounds inhibit cell mitosis by binding to the protein tubulin in the mitotic spindle and preventing polymerization into the microtubules (MTs). This mode of action is also shared with other natural agents. Therefore, there is a strong need to design and develop new natural analogs as antimitotic agents to interact with tubulin at sites different from those of *Vinca* alkaloids and taxanes [59].

**Figure 3:**
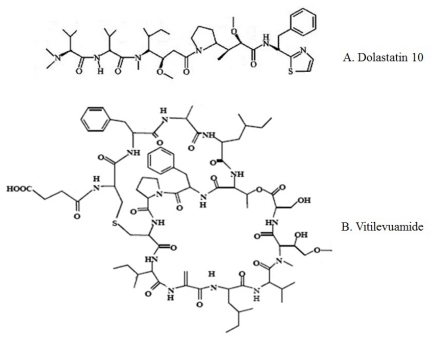
Chemical structures of major marine peptides which affect the tubulin-microtubule equilibrium: Dolastatin 10 (**A**) and Vitilevuamide (**B**).

Dolastatin 10, a linear pentapeptide containing several unique amino acid subunits (Figure 3A), was derived from the marine mollusk *Dolabella auricularia*; it is the most potent member of a large class of related peptides [60,61]. Bai *et al.* reported that Dolastatin 10 inhibited the growth of L1210 murine leukemia cells in culture [62]. Preliminary studies indicated that Dolastatin 10 causes formation of a cold-stable tubulin aggregate at higher drug concentrations. Dolastatin 10 strongly inhibits microtubule assembly, tubulin-dependent GTP hydrolysis [62], and the binding of *Vinca* alkaloids to tubulin. Dolastatin 10 prevents loss of the stabilizing effects on the colchicine binding activity of tubulin. A tripeptide segment of Dolastatin 10 also effectively inhibits tubulin polymerization and GTP hydrolysis. The tripeptide did not significantly inhibit either vincristine binding or nucleotide exchange [63].

Vitilevuamide, a bicyclic 13 amino acid peptide (Figure 3B), was isolated from two marine ascidians, *Didemnum cuculiferum* and *Polysyncranton lithostrotum*. Vitilevuamide was strongly positive in a cell-based screen for inhibitors of tubulin polymerization, displaying activity *in vivo* against P388 lymphocytic leukemia. Vitilevuamide exhibits non-competitive inhibition of Vinblastine binding to tubulin. Colchicine binding to tubulin was stabilized in the presence of Vitilevuamide. GTP binding was also found to be weakly affected by the presence of Vitilevuamide, suggesting the possibility that Vitilevuamide inhibits tubulin polymerization via an interaction at a unique site [64].

Some other peptides from marine sources, such as Diazonamide A [65,66], Scleritodermin A [67,68], Hemiasterlin [69–72], Desmethoxymajusculamide C (DMMC) [73] and Milnamide D [74] have been observed to display potent inhibition of tubulin polymerization in different cancer cells. Diazonamide A, a complex cytotoxic peptide, was isolated from the marine ascidian *Diazona angulata* [65]. Diazonamide A and the analog have a unique binding site on tubulin differing from the *Vinca* alkaloid and Dolastatin 10 binding sites. Diazonamide A and the analog bind weakly to unpolymerized tubulin but strongly to microtubule ends [65,66]. Scleritodermin A is a new cyclic peptide isolated from the lithistid sponge *Scleritoderma nodosum*. Scleritodermin A showed significant *in vitro* cytotoxicity against human tumor cell lines and inhibited tubulin polymerization [67,68]. Hemiasterlin, a natural tripeptide derived from marine sponges *Auletta* and *Siphonochalina* sp., binds to the *Vinca*-peptide site in tubulin, disrupts normal microtubule dynamics; depolymerize microtubules [69,70]. One analogue of Hemiasterlin, HTI-286, inhibits the polymerization of purified tubulin, disrupts microtubule organization in cells. HTI-286 is considered as a potent inhibitor of proliferation and has substantially less interaction with multidrug resistance protein (P-glycoprotein) than currently used antimicrotubule agents [71,72]. Desmethoxymajusculamide C (DMMC), a new cyclic depsipeptide was extracted from the Fijian Cyanobacterium *Lyngbya majuscula*. DMMC exhibited potent and selective anti-solid tumor activity against the HCT-116 human colon carcinoma cell line via disruption of cellular microfilament networks. Linearized DMMC was also evaluated in the biological assays and found to maintain potent actin depolymerization characteristics while displaying solid tumor selectivity equivalent to DMMC in the disk diffusion assay [73].

We purified an antitumor protein from the coelomic fluid of *Meretrix meretrix* Linnaeus, MML, which exhibited significant cytotoxicity to several cancer cell types, including human hepatoma BEL-7402, human breast cancer MCF-7 and human colon cancer HCT-116 cells. Further studies demonstrated that MML increased cell membrane permenbility and inhibition of tubulin polymerization [75].

## 4. Peptides that Inhibit Angiogenesis

Angiogenesis, the formation of new blood vessels, is a complex multistep process, including the destabilization of established vessel, endothelial cell proliferation, migration and new tube formation. Angiogenesis plays an important role in the growth, invasion and metastasis of most solid tumors. Both tumor growth and metastasis depend on the expansion of host vasculatures into tumors through angiogenesis [76–79]. Vascular endothelial growth factor (VEGF) and its receptor, VEGFR-2 (Flk-1/KDR), play a key role in tumor angiogenesis [80,81]. Tumor growth may be inhibited via blocking the VEGF-VEGFR-2 pathway and downstream intracellular signaling. These pathways include VEGF-induced phosphorylation of extracellular signal-regulated kinase 1/2 (ERK1/2), serine/threonine protein kinase family protein kinase B (Akt), two tumor promoters CXC chemokine Receptor (CXCR4) and Hypoxia inducible factor 1alpha (HIF1α) [82,83]. HIF1α, a subunit of HIF1 transcription factor, regulates not only adaptive responses to hypoxia, but also many cellular functions under normoxia. HIF1α induces VEGF aggregation, actions known to be important for cellular survival and endovascular differentiation [84,85].

Neovastat (AE-941) is a derivative of shark cartilage extract. Rather than being a specific monomolecular compound, AE-941 is a defined standardized liquid extract comprising the <500 kDa fraction from the cartilage of shark, *Squalus acanthias*, directly inhibits tumor cell growth and angiogenesis [86,87]. Lee *et al*. founded that Neovastat was mediated via inhibition of VEGF and HIF2 alpha pathway [88]. Mice treated with Neovastat had significantly reduced inflammatory cell count in BAL fluid. Furthermore, Mice treated with Neovastat showed significantly reduced VEGF and HIF2 alpha expression on lung tissue [88].

We purified a novel linear polypeptide with MW 15500, PG155, with potent anti-angiogenic activity from the cartilage of shark, *Prionace glauca*. The anti-angiogenic effects of PG155 were evaluated using zebrafish embryos model *in vivo*. Our study confirmed that PG155 inhibited the growth of SIV of zebrafish embryos. *In vitro* transwell experiment revealed that the polypeptide inhibited VEGF induced migration and tubulogenesis of human umbilical vein endothelial cells (HUVECs) [89].

Mycothiazole, a mixed polyketide/peptide-derived compound with a central thiazole moiety, inhibited hypoxic HIF1 signaling in tumor cells that correlated with the suppression of HIF1 target gene VEGF expression. Mechanistic studies revealed that mycothiazole selectively suppresses mitochondrial respiration at complex I (NADH-ubiquinone oxidoreductase), may serve as a valuable molecular probe for mitochondrial biology and HIF-mediated hypoxic signaling [90].

## 5. Peptides with Unknown Mechanism for Their Anti-Tumor Activity

Although a huge effort has been put on the development of anticancer agents from marine sources, this area is still a virgin field and many fewer peptides have been identified so far compared with the research on the peptides isolated from other natural sources. There are still many other peptides with unknown mechanisms for their induction of cytotoxicity, likely because they involve complicated mechanisms. A lot of peptides were isolated from ascidians, including patellamides [91–94], Styelin D [95], Eusynstyelamide [96], botryllamides A–D (1–4) [97], Lissoclinamides [46,98,99] and Mollamides [100,101], which display potent cytotoxicity, but their exact mechanism have not been well documented. Styelin D, a 32-residue *C*-terminally amidated antimicrobial peptide, isolated from blood cells of the ascidian *Styela clava* is discovered a cytotoxic peptide, was quite cytotoxic and hemolytic to eukaryotic cells [95]. Lissoclinamides (Lissoclinamides 4 and 5), a cyclic peptide isolated from the aplousobranch ascidian *Lissoclinum patella* [98], showed relevant antineoplastic as well as other pharmacological properties against human fibroblast and bladder carcinoma cell lines and normal lymphocytes [46]. The most potent is Lissoclinamide 7, containing two thiazoline rings, which rivals Didemnin B in cytotoxicity *in vitro* [99].

Sponge is another rich resource of peptides, such as Geodiamolides A–G [102,103], Orbiculamide A [104], Koshikamide B [105], Phakellistatins [106–109], Microcionamides A and B [110], Halicylindramides [111], Haligramides [112], Hemiasterlin [113], Milnamide A [114], Corticiamide A [115], Theopapuamide [116], Taumycins A [117], Koshikamide A1 [118], Koshikamide A2 [119] and Efrapeptin G [120] have been observed to display potent cytotoxicity in different cancer cells but the exact targets by these chemicals have not yet been identified. Geodiamolides A–G initially isolated from the Caribbean sponge *Geodia* sp. are a group of cytotoxic peptides in which there is three amino acids forming a cyclic peptide with a common polyketide unit [102,103]. Orbiculamide A, acyclic peptide from a marine sponge *Theonella* sp., is cytotoxic against P388 murine leukemia cells (ED_50_ = 0.34 μg/mL), but also against different melanoma cell lines [104]. Koshikamide B, a cytotoxic peptide lactone from a marine sponge *Theonella* sp., is a 17-residue peptide lactone composed of six proteinogenic amino acids, two D-isomers of proteinogenic amino acids, seven *N*-methylated amino acids, and two unusual amino acid residues. Koshikamide B exhibits cytotoxicity against P388 murine leukemia cells and the human colon tumor (HCT-116) cell line [105]. Phakellistatins, isolated from two Indo-Pacific sponges, *Phakellia costata* and *Stylotella aurantium*, are a group of proline rich cyclic heptapeptides; Phakellistatin 1, showed potent activity against P388 murine leukemia cells and other different melanoma cell lines [106]. Microcionamides A and B, new linear peptides cyclized via a cysteine moiety and isolated from the Philippine Sponge *Clathria* (*Thalysias*) *abietina*, exhibited significant cytotoxicity against the human breast tumor cells lines MCF-7 and SKBR-3 and displayed inhibitory activity against Mycobacterium tuberculosis H37Ra [110]. Similarly, several other marine anticancer peptides, including Keenamide A [121], Kulokekahilide-1 [122], Kulokekahilide-2 [123] and Scopularide A and B [124], also elicit antitumor activity via unknown mechanisms. Keenamide A, a new cytotoxic cyclic hexapeptide, was isolated from the notaspidean mollusk *Pleurobranchus forskalii*. Keenamide A exhibited significant activity against the P388, A549, MEL-20, and HT-29 tumor cell lines [121].

Some other antitumor peptides including Symplocamide A [125,126], Apratoxin D [127,128] and Mitsoamide [129] have been isolated recently from Cyanobacteria. Symplocamide A is a newly discovered 3-amino-6-hydroxy-2-piperidone (Ahp) cyclodepsipeptide, isolated from a marine cyanobacteria *Symploca* sp. in Papua New Guinea [126]. Symplocamide A is an extremely potent cytotoxin, with IC_50_ in nanomole level for H460 lung cancer and neuro-2A neuroblastoma cell lines [125]. Apratoxin D was extracted from marine cyanobacteria *Lyngbya majuscula* and *Lyngbya sordida*. Apratoxin D showed potent *in vitro* cytotoxicity against H-460 human lung cancer cells with an IC_50_ value of 2.6 nM [127]. Recently, a new cytotoxic and linear peptide was isolated from the marine cyanobacteria *Geitlerinema* sp. [129]. Additionally, two novel cyclodepsipeptides, Scopularides A and B were found in the fungus *Scopulariopsis brevicaulis* and activity of Scopularides against several tumor cell lines was significant at 10 μg/mL [124]. However, the exact mechanisms by these peptides have not yet been identified.

## 6. Conclusions

The discussed marine peptides and their mode actions are summarized in Table 1. However, a lot of marine peptides display antitumor activity via multiple targets. Dolastatin 10 not only inhibits microtubule assembly, but also induces apoptosis associating with a decrease in Bcl-2 level and an increase in p53 expression in the lymphoma cell line [130]. Aplidine’s mechanism of action involves several pathways, including cell cycle arrest, inhibition of protein synthesis and anti-angiogenic activity [131].

Peptide compounds reviewed here are obtained from very different marine organisms with different mechanism on their antitumor activity. Because of the peculiarities of the life in the sea, many of these molecules can be found only in a single source For example, Jaspamide (Jasplakinolide) is only found in marine sponge Jaspis species [28–30], and Trididemnum genus of marine tunicate is the only source for Didemnin B [44–49]. Although the underling mechanism of source specificity of the marine peptides is unclear, it is conceivable that the special environment of marine offers the diversity of marine natural products. It is also possible that the organisms, which may contain the peptides, have not been found since the study of marine peptides is still in its infancy. Compared with the peptides found from other sources, there is more diversity on the style/classes of marine peptides; more cyclic peptides or depsipeptide were found in marine organism. These marine peptides seem to be very useful and promising for biomedical research. There is no doubt that the diversity of marine peptides in its structure and mode of action provide a rich source for the design of very specific and potent new pharmaceuticals for a wide variety of diseases.

## Figures and Tables

**Table 1:** A summary of marine peptides and their mode of actions.

Name of peptides	Sources	Class/types	Mode of action and references
Jaspamide (Jasplakinolide)	Marine sponge, *Jaspis johnstoni*	Cyclic depsipeptide	Caspase-3 activation, decreasing in Bcl-2 protein expression [28–30]
Somocystinamide A (ScA)	*Lyngbya majuscula*/*Schizothrix* sp. assemblage of marine cyanobacteria	Lipopeptide	Caspase-8 activation [31,32]
*C*-phycocyanin (*C*-PC)	Cyanobacteria *Agmenellum quadruplicatum, Mastigocladus laminosus*, S*pirulina platensis*	Tetrapyrrole-protein complex	Caspases-dependent apoptosis [34]
Aplidine (dehydrodidemnin B, DDB, Aplidin)	Tunicate, *Aplidium albicans*	Cyclic depsipeptide	JNK and p38 MAPK phosphorylation activation [37–39]
Didemnin B	Tunicate, *Trididemnum solidum*	Cyclic depsipeptide	Apoptosis, but unclear [44–49]
Sansalvamide A	Marine fungus	Cyclic depsipeptide	Apoptosis, but unclear [53,54]
Cycloxazoline	Marine ascidian, *Lissoclinum bistratum*	Cyclic depsipeptide	Apoptosis, but unclear [55,56]
Virenamides A–C	Didemnid ascidian, *Diplosoma virens*	Linear tripeptides	Apoptosis, but unclear [57]
Dolastatin 10	Marine mollusk, *Dolabella auricularia*	Linear peptide	Microtubule assembly Inhibition [60,62]
Vitilevuamide	Marine ascidians, *Didemnum cuculiferum* and *Polysyncranton lithostrotum*	Bicyclic peptide	Tubulin polymerization inhibition [64]
Diazonamide	Marine ascidian, *Diazona angulata*	Macrocyclic peptide	Tubulin polymerization inhibition [65,66]
Scleritodermin A	Lithistid sponge, *Scleritoderma nodosum*	Cyclic peptide	Tubulin polymerization inhibition [67,68]
Hemiasterlin	Marine sponges, *Auletta* sp. and *Siphonochalina* sp.	Tripeptide	Tubulin polymerization inhibition [70–72]
DMMC	Cyanobacterium *Lyngbya majuscula*	Cyclic depsipeptide	Tubulin polymerization inhibition [73]
MML	Coelomic fluid, *Meretrix meretrix*	Protein	Tubulin polymerization inhibition [75]
Neovastat (AE-941)	Shark cartilage, *Squalus acanthias*	Extract < 500 kDa	VEGF and HIF2 alpha pathway inhibition [86–88]
PG155	Shark cartilage, *Prionace glauca*	Polypeptide	VEGF induced angiogenesis inhibition [89]
Styelin D	Ascidian, *Styela clava*	*C*-terminally amidated peptide	Unknown [95]
Lissoclinamides	Aplousobranch ascidian, *Lissoclinum patella*	Cyclic peptide	Unknown [46,98,99]
Geodiamolides A–G	Caribbean sponge, *Geodia* sp.	Cyclic peptide	Unknown [102,103]
Orbiculamide A	Marine sponge, *Theonella* sp.	Cyclic peptide	Unknown [104]
Koshikamide B	Marine sponge, *Theonella* sp.	Peptide lactone	Unknown [105]
Phakellistatins	Marine sponges	Cyclic heptapeptides	Unknown [106–109]
Microcionamides A and B	Philippine Sponge, *Clathria (Thalysias) abietina*	Linear peptides	Unknown [110]
Keenamide A	Notaspidean mollusk, *Pleurobranchus forskalii*	Cyclic hexapeptide	Unknown [121]
Scopularides A and B	Fungus *Scopulariopsis brevicaulis*	Cyclodepsipeptide	Unknown [124]
Symplocamide A	Marine cyanobacteria *Symploca* sp.	Cyclodepsipeptide	Unknown [125,126]
Apratoxin D	Marine cyanobacteria *Lyngbya majuscula* and *Lyngbya sordida*	Macrocycle peptide	Unknown [127,128]
Mitsoamide	Marine cyanobacteria *Geitlerinema* sp.	Linear peptide	Unknown [129]
